# Vaccination against neoantigens induced in cross-priming cDC1 in vivo

**DOI:** 10.1007/s00262-023-03597-y

**Published:** 2024-01-17

**Authors:** Emily S. Clark, Ana Paula Benaduce, Wasif N. Khan, Olivier Martinez, Eli Gilboa

**Affiliations:** 1 Department of Microbiology and Immunology, Miller School of Medicine, University of Miami, 1550 NW 10th Avenue Medical Campus, Papanicolaou Building 257, Miami, FL 33136 USA; 2 Sylvester Comprehensive Cancer Center, Miller School of Medicine, University of Miami, Miami, FL USA; 3 Present Address: Department of Biological Sciences, Florida International University, Miami, FL USA; 4 Present Address: Mnemo Therapeutics, Paris, France

**Keywords:** Cancer vaccines, Transporter associated with antigen presentation (TAP), Dendritic cells, siRNA, Neoantigens

## Abstract

The conventional type 1 dendritic cells (cDC1) play a pivotal role in protective immunity against pathogens and cancer. However, their low frequency in the blood and tissues limits their use in immune therapy. We have recently described a method to vaccinate against neoantigens that are induced in tumor cells by targeted delivery of a TAP siRNA to dendritic cells using a TLR9 binding CpG oligonucleotide. Since TLR9 is also expressed in immune suppressive myeloid populations TLR9 targeting could reduce the effectiveness of this approach. Here, we describe a modular multivalent antibody platform to target the TAP siRNA to resident Clec9a expressing cDC1 and show that it leads to selective and sustained TAP downregulation in cDC1 and inhibits tumor growth in mice more effectively than CpG targeted siRNA. To induce DC maturation an agonistic CD40 antibody was administered to the siRNA treated mice. To obviate the need for a second drug formulation and reduce the risk of toxicity, we exploited the multivalent nature of this targeting platform to co-deliver the TAP siRNA and a DC maturation agent, a CpG containing oligonucleotide, to cDC1 in vivo and show that it was more effective than Clec9a targeting of TAP siRNA in combination with CD40 antibody. This study describes a way to manipulate the function of cDC1 cells in vivo using a broadly applicable antibody-based targeting platform to deliver multiple biological agents to specific cells in vivo to potentiate (immune) therapy and to probe the biology of specific cell types in their natural settings.

**Supplementary Information:**

The online version contains supplementary material available at 10.1007/s00262-023-03597-y.

## Introduction

The cDC1 subset of dendritic cells (DC) is superior in cross-presenting soluble or cell derived antigens for MHC class I presentation required for the generation of CD8 + T cell responses and plays a pivotal role in protective immunity against pathogens and cancer (reviewed in [1–4]). Tumor resident cDC1 is also the main producers of CXCL9 and CXCL10 chemokines that promote the recruitment of CD8 + T cells to the tumor microenvironment [2, 4]. cDC1 has been proposed to serve as the next generation of DC-based vaccines [1, 4, 5]. However, their low frequency in the blood and tissues is a barrier to their use in immune therapy and in the ability to study their biology. To circumvent this limitation, studies have sought to develop methods of manipulating cDC1 in vivo by targeted delivery of antigens to stimulate protective immunity. Expression of the C-type lectin Clec9a/DNGR-1 is largely restricted to cDC1 [3, 6]. Targeting antigens to cDC1 in the form of peptide epitopes or whole antigen covalently linked to Clec9a Ab elicited CD8 + cytotoxic T cell responses as well as humoral and CD4 + T cell responses in mice [6], reduced viremia [7], and inhibited tumor growth [8]. Clec9a targeting was comparably or more effective than targeting to receptors expressed more broadly on dendritic cells like DEC205 or mannose receptor [9–11].

We have recently described a new vaccination concept targeting a common set of antigens that are induced experimentally in tumor cells by downregulation of the transporter associated with antigen processing (TAP), termed T cell epitopes associated with impaired peptide processing (TEIPP) [12]. TAP was downregulated using an siRNA that was targeted to tumor cells in mice by conjugation to a nucleolin (Nucl)-binding oligonucleotide aptamer, a broad-spectrum tumor targeting ligand [13], and vaccinated against the induced TAP TEIPP by targeted delivery of the TAP siRNA to resident dendritic cells using a short Toll-like receptor 9 (TLR9) binding CpG containing oligonucleotide. Studies in mice have demonstrated the feasibility, broad applicability, and potency of this vaccination strategy to elicit T cell mediated antitumor immunity in the absence of measurable toxicity [14]. One limitation of this approach is that TLR9 is expressed on all DC subsets in mice as well as in B cells, macrophage/monocyte subsets, neutrophils and eosinophils, including myeloid derived suppressive cells (MDSC), M2 type macrophage and incompletely matured DC [15–17] that could lead to the presentation of the induced neoantigens by immune suppressive antigen presenting cells. In this study, we describe a way to limit TAP downregulation to resident cDC1 in mice and confine activation to cDC1 that present the TAP downregulation induced antigens.

## Materials and methods

### Mice

All animal work was conducted under the approval of the University of Miami Institutional Animal Care and Use Committee (IACUC) in accordance with federal, state, and local guidelines. Female, 10–12-week-old C57Bl/6 mice were used for all studies and purchased from The Jackson Laboratory.

### Generation of the Clec9a antibody-oligonucleotide (ODN) conjugate

Antibody conjugations were carried out using a modified protocol from Vector Laboratories for protein-oligonucleotide conjugation. All reagents, unless otherwise indicated, were purchased from Vector Laboratories. The antibody-oligonucleotide calculator (https://vectorlabs.com/wp-content/uploads/2022/11/VL_A-9002-001.UserGuide.LBL-01990.pdf) available from Vector Laboratories was used throughout the protocol. Conjugation was carried out in two steps. First, a 5′ amino-modified ODN was labeled with S-4 formylbenzamide (4-FB). Next, the 4-FB modified ODN was covalently linked to a S-Hynic labeled antibody molecule using an analine catalyst to generate a stable antibody-ODN conjugate. To label the ODN with 4-FB, the 5′ amino-modified ODN was resuspended in 1× Modification Buffer at a concentration of 0.5 OD/µL, diluted 1:200 in H_2_O and the concentration (OD_260_/µL) determined using a NanoDrop. This value, along with the total volume, was entered into the calculator. The ODN, the calculated volume of andrydrous DMF, and the resuspended S-4-FB were combined and incubated for 2 h at RT on a rotator. The 4-FB ODN was purified and desalted twice into 1 × Conjugation Buffer using a 7 K MWCO Zeba Column (Thermo Scientific) according to the manufacturer’s instructions. Following purification, the ODN concentration was determined, and it was aliquoted and stored at − 80 °C. Aiming at conjugating 8–10 ODN molecules per antibody, the antibody (*InVivoMAb* anti-mouse Clec9A (CD370) Clone 7H11, or rat IgG1 isotype control clone HRPN; BioXCell) was desalted into 1 × Modification Buffer using Amicon Ultra 30 K MWCO centrifugal filters (Millipore). Three buffer changes were done according to the manufacturer’s instructions. The antibody was collected, and the protein concentration was measured using a Nanodrop and adjusted to be not more than 2 mg/ml. S-Hynic was resuspended in anhydrous DMF to label the antibody with a 50-molar excess of S-Hynic (µg IgG = 6.6 pmol) and incubated for 2.5 h at RT on a rotator. Excess S-Hynic was removed by buffer exchange into 1X Conjugation Buffer using Amicon filters as described above. The S-Hynic modified antibody was incubated with 10-molar excess 4-FB modified ODN in 10X Catalyst Buffer. Following incubation, the antibody-ODN was desalted into PBS (no Ca^++^ or Mg^++^, Gibco) using the Amicon filters as described above. The antibody concentration was measured using the Pierce BCA protein assay (Thermo Scientific) and stored at 4 °C for not more than 1 month. For each experiment, complementary ODN (cODN) modified siRNA and/or CpG ODNs were hybridized to the antibody conjugated ODNs at a molar ratio of 1:1 at 41 °C for 15 min in PBS with divalent cations to yield 8–10 ODN/antibody molecule.

To determine that ODNs were conjugated to the antibody and that excess free ODNs were removed, 1 mg of antibody-ODN conjugate was subjected to 2% agarose gel electrophoresis in TBE buffer, stained with ethidium bromide and visualized with UV. To determine whether ODN conjugation has affected antibody binding, the antibody-ODN conjugates were incubated with Clec9a-Fc-beads and checked for fluorescence saturation by flow cytometry. To prepare the beads, 10 µl Protein A DynaBeads (Thermo Scientific) were washed three times with bead buffer (20 mM Hepes, pH 7.4, 10 mM NaCl, 2 mM CaCl_2_, 2 mM MgCl_2_, 0.02% azide, 0.01% Tween-20). The washed beads were incubated with 10 µg of murine rClec-9A-Fc chimeric protein (R&D) in 100 µL bead buffer for 1 h at RT with rotation and washed as before followed by incubation with 10 µg IgG isotype antibody. Finally, the beads were washed as before and stored in 1 mL of bead buffer at 4 °C until use. To estimate the number of ODNs conjugated per antibody, 1 µg of Clec9A-ODN was incubated with increasing molar excess of cODN-AlexaFluor-647. Following hybridization, the reaction mixture was incubated with 10 µL of Clec9a DynaBeads for 10 min at RT. Beads were washed in mL FACS Buffer (PBS + 2% FBS) and analyzed by flow cytometry as detailed below. An unrelated antibody (mPD-1 clone RMP1-14; BioXCell)-ODN was used as a negative control for specificity of the Clec9a-ODN antibody to the bead.

### ODN sequences

The following ODN sequences were used in the study as indicated in figure legends. All ODNs were either purchased from Integrated DNA Technologies or Trilink BioTechnologies, Inc. Where indicated, 2’* O*-methylated (m) pyrimidines or phosphorothioate (*) modified oligos were used to increase stability. Duplexed Tap 2 siRNA (Sense: 5′-GmCmUGmCAmCAmCGGmUmUmCAGAAmU; Antisense: 5′-AUUCUGAACCGUGUGCAGCmUmU) or Scrambled Control (Sense: 5′-mUAAAGAAmCmCAmUGGmCmUAAmCmC; Antisense: 5′ GGUUAGCCAUGGUUCUUUAmUmU) were delivered to target cells by conjugation to Nucleolin aptamer, CpG, or antibody. The Nucleolin aptamer (5′-GGTGGTGGTGGTTGTGGTGGTGGTGG) extended at the 3′ end with a 12-carbon spacer followed by a linker (5′-GmUmAmCAmUmUmCmUAGAmUAGmCmC) was annealed to the 5′ end of Tap 2 or control siRNA using a 3′ complementary linker sequence separated from the sense sequence by a nine-carbon spacer and was used to deliver Tap 2 siRNA to RMA cells. CpG 1668 (5′-T*C*C*A*T*G*A*C*G*T*T*C*C*T*G*A*T*G*C*T*) extended at the 3′ end with a 12-carbon spacer followed by the linker: 5′-mCGAGGmCmUAmUmCmUAGAAmUGmUAmC was annealed to a complementary sequence on the Tap 2 siRNA as above and used to deliver Tap 2 siRNA to TLR9 expressing cells. Antibodies were used to deliver Tap 2, control scrambled siRNA, CpG, or control CpG (5′-T*C*C*A*T*G*A*G*C*T*T*C*C*T*G*A*G*C*T*T*) ODNs by annealing to the ODN (5′-/5AmMC6/rAmUrAmGmUrAmCrAmUmUmCmUrArGrAmUrArGmCmC) labeled antibody with a 3′ complementary linker separated from the sequence by a nine-carbon spacer and was used to deliver Tap 2 siRNA or control sequence to Clec9A-expressing cells. Annealing was carried out at equimolar ratio of the complementary sequences in PBS with Ca^2+^ Mg^2+^ for 5 min at 85 °C in a heat block and allowed to cool to room temperature and stored at − 80 °C until use.

### Measuring TAP expression in cells by intracellular flow cytometry

Mice were euthanized, and spleens were removed at timepoints indicated in figure legends. A single cell suspension of the spleen was prepared and filtered through a 70 µm nylon filter. Red blood cells were lysed using ACK Buffer (ThermoFisher), and cells were resuspended in FACS Buffer. FcR was blocked (Fc Block; BD Biosciences), and splenocytes were stained with antibody cocktail diluted in Brilliant Stain Buffer (BD Biosciences) for 30 min at 4 °C and washed with PBS. Cells were incubated with Live-Dead Blue (ThermoFisher Scientific) for 15 min at RT, washed twice with PBS and fixed with BD Cytofix/Cytoperm according to the manufacturer’s instructions. Intracellular staining was performed in two steps. First, cells were stained with anti-Tap2 antibody for 30 min at RT, washed with Perm Wash Buffer (BD), and then stained with anti-rabbit AlexaFluor647 for 30 min at RT. Cells were washed a final time and then collected using a Cytek Aurora spectral analyzer flow cytometer (Flow Cytometry Shared Resource; UMiami Sylvester Cancer Center). Data was rendered and analyzed using FlowJo software (TreeStar). Multicolor flow cytometry was performed with the following antibodies: CD3e-AlexaFluor488 (145-2C11), CD49b-AlexaFluor488 (DX5), Ly6G-AlexaFluor488 (1A8), MHCII(I-A/I-E)-APC-Fire750 (M5/114.15.2), XCR1-PE (ZET), CD11b-PE-Cy5 (M1/70), Ly6C-BV605 (HK1.4), CD172a (SIRPa)-PE-Cy7 (P84), and CD317-BV711 (927) from BioLegend; F4/80-APC-R700 (T45-2342), Siglec-H-BV421 (440c), CD19-BUV805 (1D3), and CD11c-BB700 (N418) from BD Biosciences; and rabbit polyclonal anti-Tap2 (PA5-37,414) and anti-rabbit AlexaFluor647 (A21443) from ThermoFisher Scientific.

### Tumor models

RMA (Tap2 sufficient) and RMA-S (Tap2 deficient) cell lines were used for all tumor studies and have been described [13, 14]. Cells were cultured in IMDM supplemented with 10% FBS (Cytiva), Pen/Strep, sodium pyruvate, non-essential amino acids, and 2-mercaptoethanol (all from Gibco). For tumor cell injection, cells were collected and washed twice in PBS.

#### RMA-S T lymphoma model

Seven to 9-week-old female C57Bl/6 mice were injected s.c. in the right flank with 4 × 10^5^ tumor cells. Four days after injection, mice were treated either with a single dose of 0.75 nmol CpG-siRNA conjugate (1.3 mg/kg) injected subcutaneously close to the inguinal lymph node in the right flank or with three doses of 100 µg of antibody-siRNA/ODN injected intraperitoneally daily.

#### RMA T lymphoma model

Seven to 9-week-old female C57BL/6 mice were injected s.c. with 5 × 10^4^ RMA tumor cells. Four days after injection, mice were treated either with a single dose of 0.75 nmol CpG-siRNA conjugate (1.3 mg/kg) injected subcutaneously close to the inguinal lymph node in the right flank or with three doses of 100 µg of antibody-siRNA conjugate injected intraperitoneally. When palpable tumors with volume of ~ 10–25 mm^3^ were measured, Nucl-siRNAs were administered intraperitoneally (i.p.) at 1 nmol (1.75 mg/kg). This was repeated two additional times 3 days apart.

For both models, tumor length and width were measured using digital calipers. Tumor volume reaching 1000 mm^3^ or tumor ulceration was used as the experimental endpoint.

### Statistical analysis

When variables studied were normally distributed, statistical analysis of multiple comparisons was performed using one-way ANOVA with Tuckey or Dunnet post-test. To compare the mean differences between groups that have been split into two independent variables (treatment/number of doses), analyses were performed using two-way ANOVA. Nonparametrical methods were applied for not normally distributed variables. For these statistical analyses, multiple comparisons were performed using Kruskal–Wallis with Dunn post-test, and comparisons between just two groups were performed using Mann–Whitney *U*-test. Significance of overall survival was determined via Kaplan–Meier analysis with log-rank (Mantel–Cox) analysis. All statistical analyses were performed with Graphpad Prism 6 and 7 (GraphPad). Error bars show standard error of the mean (SEM) and *p* < 0.05 was considered statistically significant.

## Results

### Selective downregulation of TAP in cDC1

**Fig. 1:**
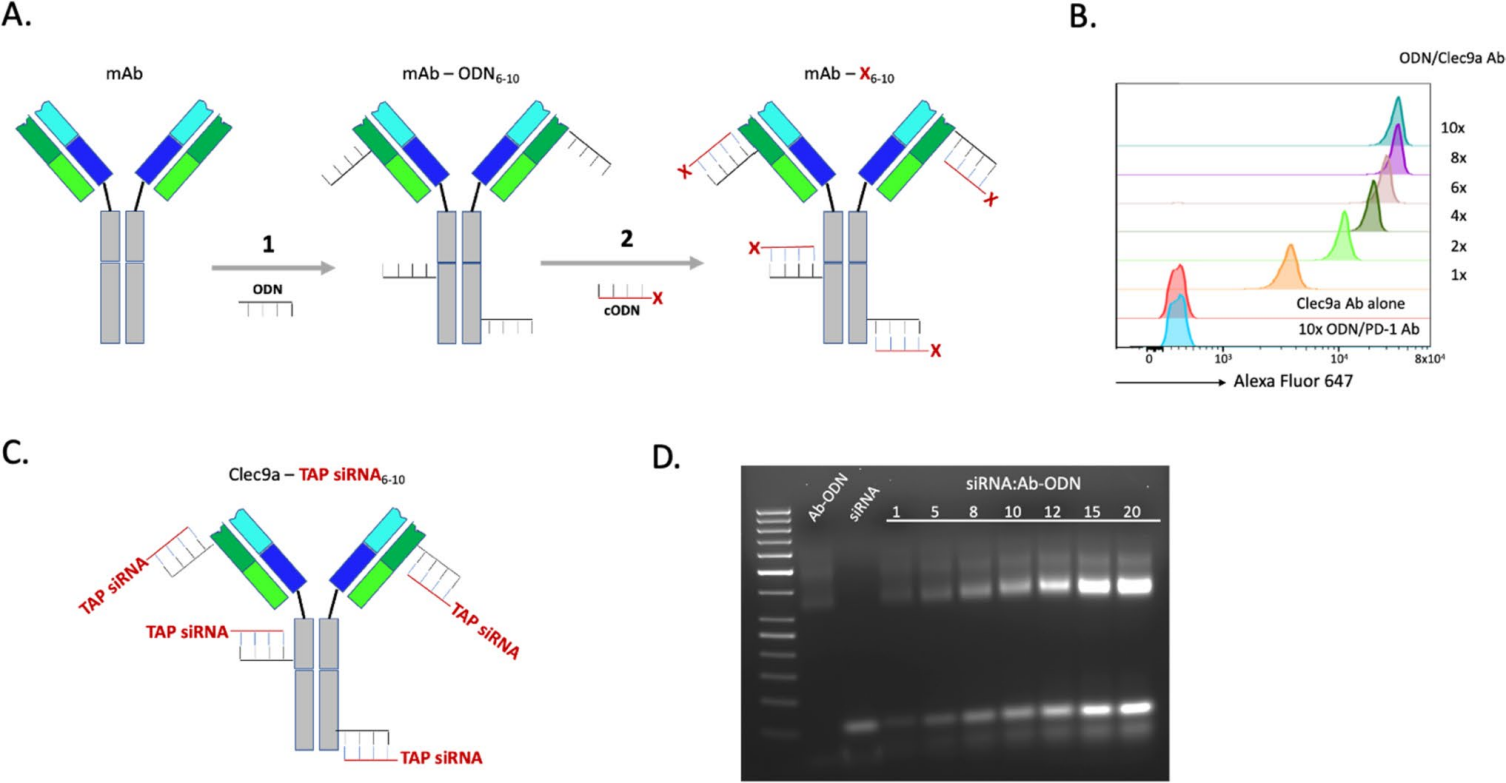
Conjugation of TAP siRNA to Clec9a antibody. **a** A modular multivalent antibody-based targeting platform. Step 1, A 19 nt modified ribooligonucleotide (ODN) is conjugated to 8–10 alpha amino groups of about 40 accessible lysines using a stable bis-aryl hydrazone bond (mAb-ODN). Step 2, A complementary ODN (cODN) is attached to the targeted agent (X) and hybridized to the ODN-modified antibody in aqueous solution like PBS at 1:1 ratio. The ODN-modified antibody is aliquoted and stored at − 80 °C. b. Valency of the antibody-ODN. The average number of ODNs conjugated to the antibody was determined by hybridization of a fluorophore-labeled cODN and flow cytometry. Clec9a antibody-TAP siRNA. 8–10 copies of a murine TAP siRNA with a cODN attached to the 3′ end of the sense strand by cosynthesis were hybridized to the ODN-modified Cle9a antibody. **d** Monitoring hybridization reaction by agarose gel analysis. ODN conjugated Cle9a antibody (Ab-ODN) was hybridized with TAP siRNA (siRNA) at increasing molar ratios of siRNA to ODN, run on a 2% agarose gel and visualized by UV. siRNA binding was saturated between 10–12 siRNA molecules per antibody. The antibody-siRNA conjugate is aliquoted and can be stored at 4 °C for at least 30 days

To downregulate TAP in resident cDC1 in mice with a TAP specific siRNA, we used a modular multivalent antibody targeting platform (Fig. 1a) whereby 8–10 copies of a 19 nt long 2’-*O*-methyl (2’OMe)-modified ODN are chemically conjugated to a Clec9a antibody (Step 1). A fluorophore-labeled complementary ODN was used to determine the average number of ODNs conjugated per antibody (Fig. 1b). In Step 2, the cargo (Fig. 1a, X) is conjugated to the ODN-modified antibody by hybridization via an attached complementary sequence (cODN) resulting in a multivalent configuration whereby the antibody delivers multiple cargo molecules to the targeted cell. 8–10 copies of a TAP or control siRNA were conjugated to a Clec9a antibody via complementary sequences engineered at the 3′ end of the siRNA sense strand (Fig. 1c). Hybridization of the siRNA to the ODN-modified Clec9a antibody was monitored by agarose gel analysis (Fig. 1d).

**Fig. 2:**
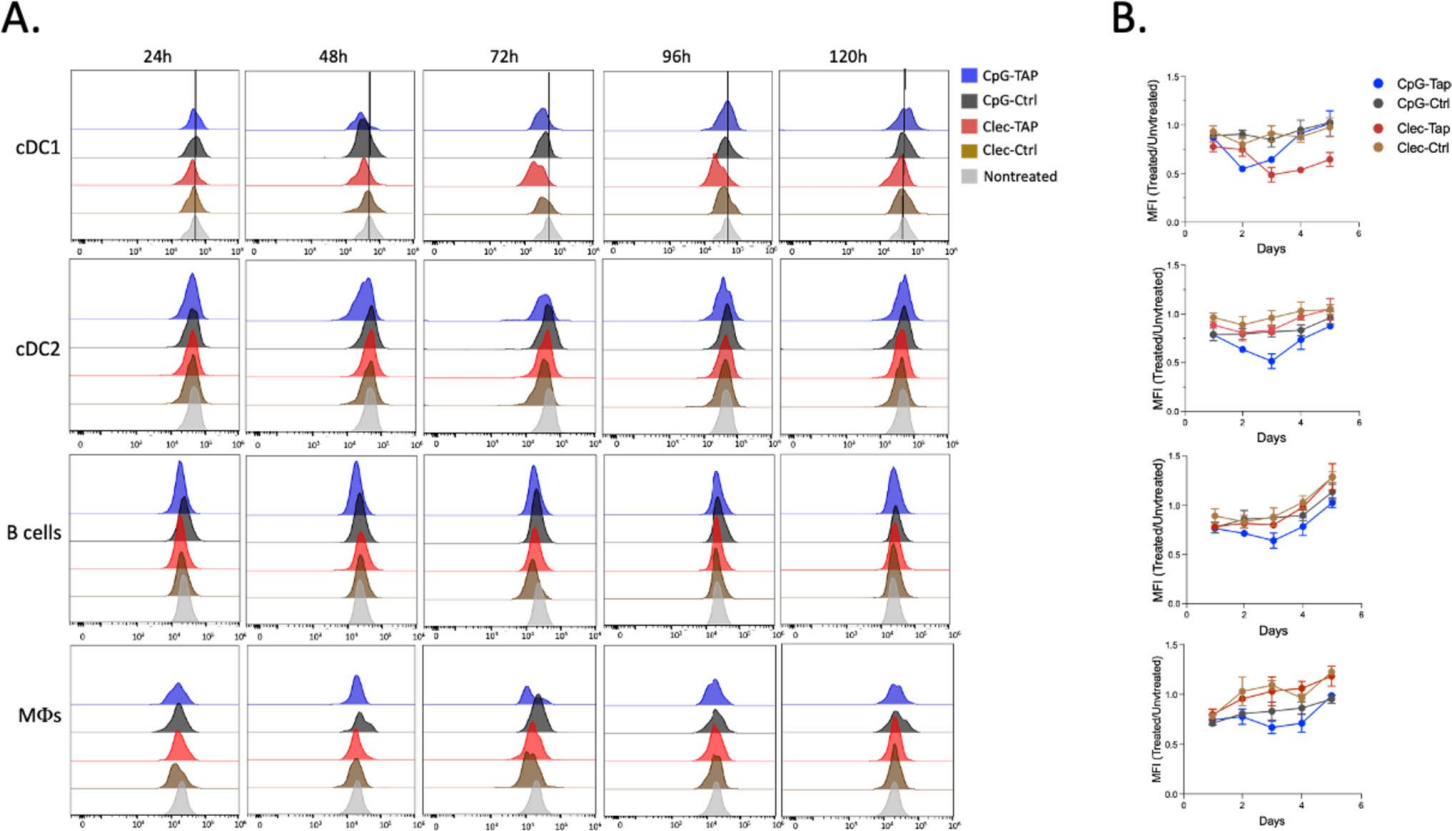
Cle9a targets TAP siRNA to resident cDC1 in mice. CpG ODN and Clec9a antibody conjugated to either TAP or control siRNA were administered to mice by intraperitoneal injection, splenocytes isolated, and TAP expression in cDC1, cDC2, B cells and macrophages was monitored over time by multiparameter flow cytometry (Fig. S1). **a** Individual mouse. **b** Time course showing TAP expression relative to untreated mice, average of three mice per group. Statistical significance of *p* < 0.05 was reached in cDC1 CpG-TAP versus CpG-Ctrl days 2 and 3, Clec-TAP-Clec-Ctrl days 3, 4 and 5, Clec-TAP versus CpG-TAP days 3, 4, and 5; cDC2 CpG-TAP versus CpG-Ctrl days 2 and 3, CpG-TAP versus Clec-TAP days 2 and 3; B cell CpG-TAP versus CpG-Ctrl day 3; Macrophages, no statistical differences except a trend in CpG-TAP versus CpG-Ctrl days 3 and 4

Downregulation of TAP was evaluated by flow cytometry in cDC1, cDC2, B cells and macrophages isolated from the spleens of mice treated with Clec9A antibody or CpG ODN targeted TAP siRNA. Figure 1a shows the results from a representative mouse. For example, 72 and 96 h post-treatment with Clec9a targeted TAP siRNA a significant downregulation of TAP in cDC1 cells can be seen when compared to treatment with Clec9A antibody targeting a control siRNA, whereas treatment with CpG ODN targeted TAP siRNA downregulation was less pronounced and more transient, seen at 72 h but not at 96 h post-treatment. TAP downregulation averaging three mice per group is shown in Fig. 2b. CpG ODN targeted TAP siRNA led to transient TAP downregulation in all subsets analyzed, consistent with the broad distribution of TLR9 in DC, B cells, and monocyte/macrophages [15–17]. In contrast, Clec9A antibody targeted TAP siRNA led to TAP downregulation in cDC1, but not in cDC2, B cells, or macrophages, consistent with the selective expression of Clec9a in cDC1. Clec9a mediated TAP downregulation was also more sustained than CpG ODN mediated TAP downregulation, conceivably reflecting the longer half-life of antibodies in the circulation. Isotype antibody conjugated TAP siRNA failed to downregulate TAP in cDC1 cells (data not shown). Taken together this experiment shows that Clec9a Ab targets its attached TAP siRNA cargo to cDC1 in vivo that results in TAP downregulation.

### Inhibition of tumor growth

**Fig. 3:**
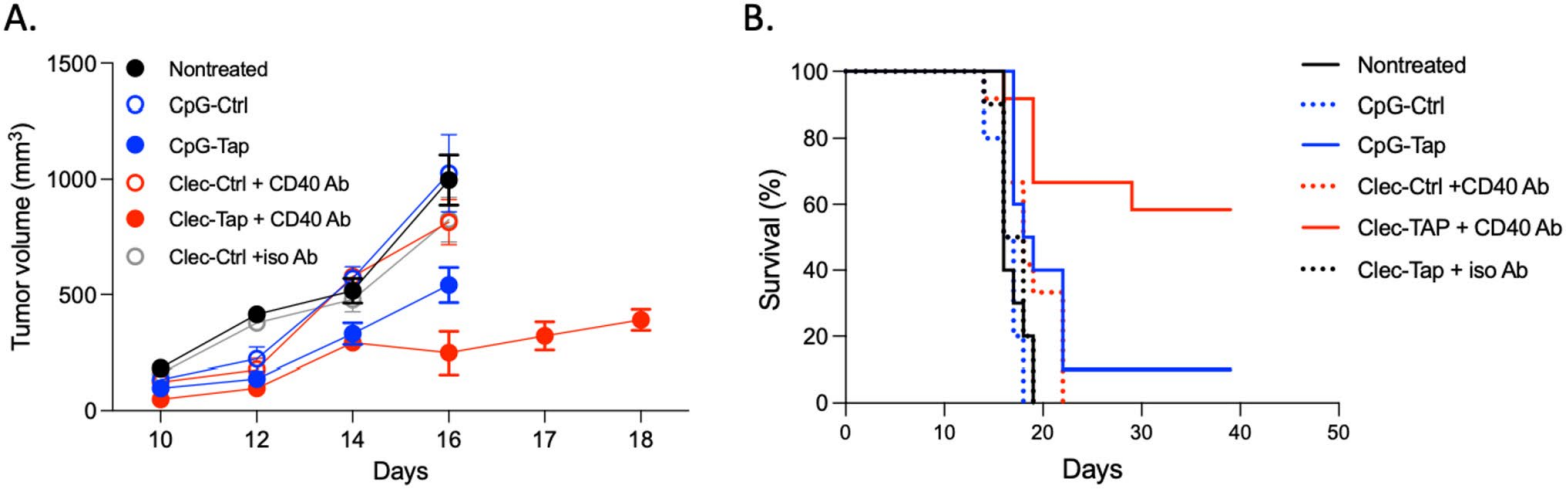
Treatment of TAP-deficient RMA-S tumor bearing mice with Clec9a targeted TAP siRNA inhibits tumor growth. C57Bl/6 mice were implanted subcutaneously with RMA-S tumor cells and four days later CpG ODN and Cle9a antibody conjugated to TAP or control siRNAs were administered intraperitoneally (10 mice/group). Where indicated, an agonistic CD40 antibody or isotype control antibody was administered intraperitoneally to mice treated with Cle9a antibody. **a** Tumor volume. Measurements were terminated when two mice were sacrificed because tumors have reached maximum allowable volume. CpG-TAP versus CpG-Ctrl* p* < 0.05, Cle9-TAP versus Clec-Ctrl, *p* < 0**.**005, Cle9-TAP versus CpG-TAP *p* < 0.05, **b** Survival. CpG-TAP versus CpG-Ctrl *p* < 0.05, Cle9-TAP versus CpG-TAP, *p* < 0.005

We next tested whether treatment of mice with TAP siRNA targeted to cDC1 can inhibit tumor growth. Figure 3 shows that treatment of mice bearing the TAP-deficient RMA-S tumor that naturally present TAP TEIPP with Clec9a antibody targeted TAP siRNA inhibited tumor growth, reducing the rate of tumor growth (Fig. 3a) and extending the survival of the treated animal (Fig. 3b). Figure 3 also shows that Clec9a targeting of the TAP siRNA was more effective than CpG ODN targeting.

**Fig. 4:**
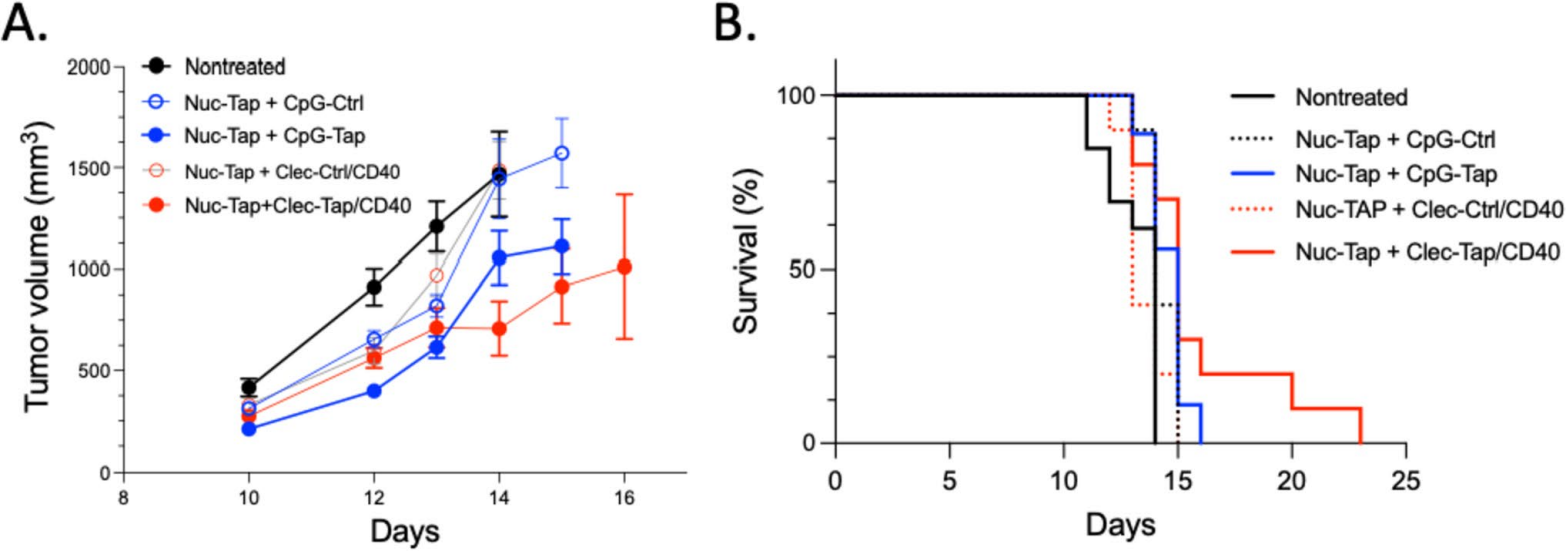
Treatment of TAP sufficient RMA tumor bearing mice with Clec9a targeted TAP siRNA inhibits tumor growth. As in the legend to Fig. 3 except that mice were also treated with nucleolin aptamer targeted TAP siRNA). Panel A CpG-TAP versus CpG-Ctrl and Clec-TAP versus Clec-Ctrl days 14 and 15* p* < 0.05. Panel B Clec9-TAP versus Clec-Ctrl or CpG-TAP *P* < 0.05. CpG-TAP versus CpG-Ctrl, ns

To test whether Clec9a-TAP siRNA can also inhibit TAP in TAP sufficient RMA tumor cells, expression of TAP was partially and transiently reduced in the tumor bearing mice by treatment with a TAP siRNA targeted to the RMA tumor cells by conjugation to a nucleolin binding DNA aptamer (Nucl-TAP siRNA) as previously described [13]. Figure 4 shows that treatment of the TAP sufficient RMA cells with Clec9a antibody targeted TAP siRNA inhibited tumor growth provided mice were also treated with Nucl-TAP siRNA. Clec9a targeting was more effective than CpG ODN targeting, but the difference was less pronounced than seen in RMA-S tumor bearing mice (Fig. 3). Taken together, these experiments show that Cle9a antibody targeted delivery of TAP siRNA to cDC1 cells in mice inhibits tumor growth and is more effective than CpG ODN targeting. This difference could reflect the sustained TAP downregulation in cDC1 cells (Fig. 2b) and/or the restricted expression pattern of Clec9a largely confined to cDC1 (Fig. 2) precluding the presentation of the induced TAP TEIPP by immune suppressive myeloid cells like MDSC, M2 macrophages or incompletely matured DC.

### Targeted co-delivery of maturation stimuli

**Fig. 5:**
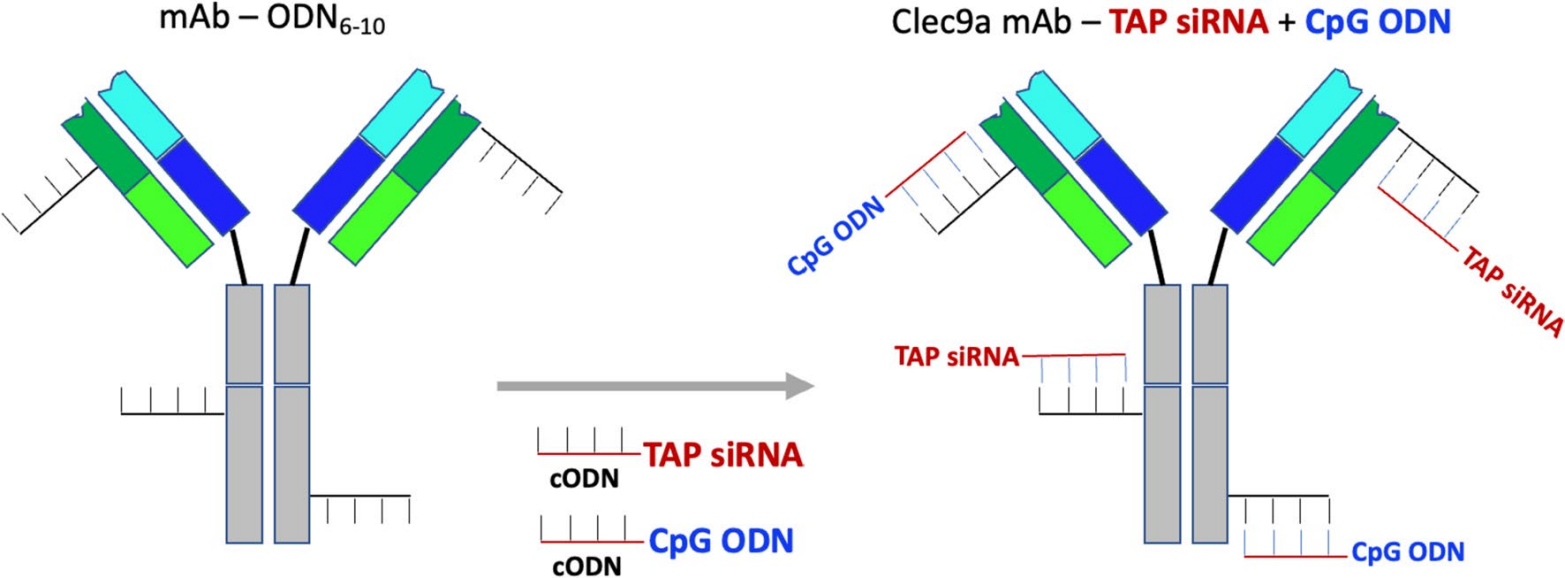
Conjugation of CpG ODN and TAP siRNA to the Clec9a antibody. An equimolar mixture of CpG ODN and TAP siRNA was hybridized to the oligo-modified Clec9a antibody as described in Fig. 1, averaging four to five CpG ODNs and TAP siRNAs per antibody

While Clec9a targeting provides more specificity than CpG ODN targeting (Fig. 2), unlike engagement of TLR9 by CpG ODNs cross-linking of TAP siRNA with the Cle9a antibody does not induce DC activation/maturation which is required to elicit an effective immune response against the antigens presented by the cDC1. Activation/maturation stimuli were, therefore, provided separately by co-treatment with an agonistic CD40 antibody [18, 19]. In patients, treatment with systemically administered CD40 antibody was associated with immune related adverse effects [20]. To dispense with the need of using the agonistic CD40 antibody and reduce the risk of toxicity, we conjugated both the TAP siRNA and a CpG ODN to the Clec9a antibody by hybridization via common complementary sequences as shown in Fig. 5, thereby limiting activation to cDC1 that present the induced antigens.

**Fig. 6:**
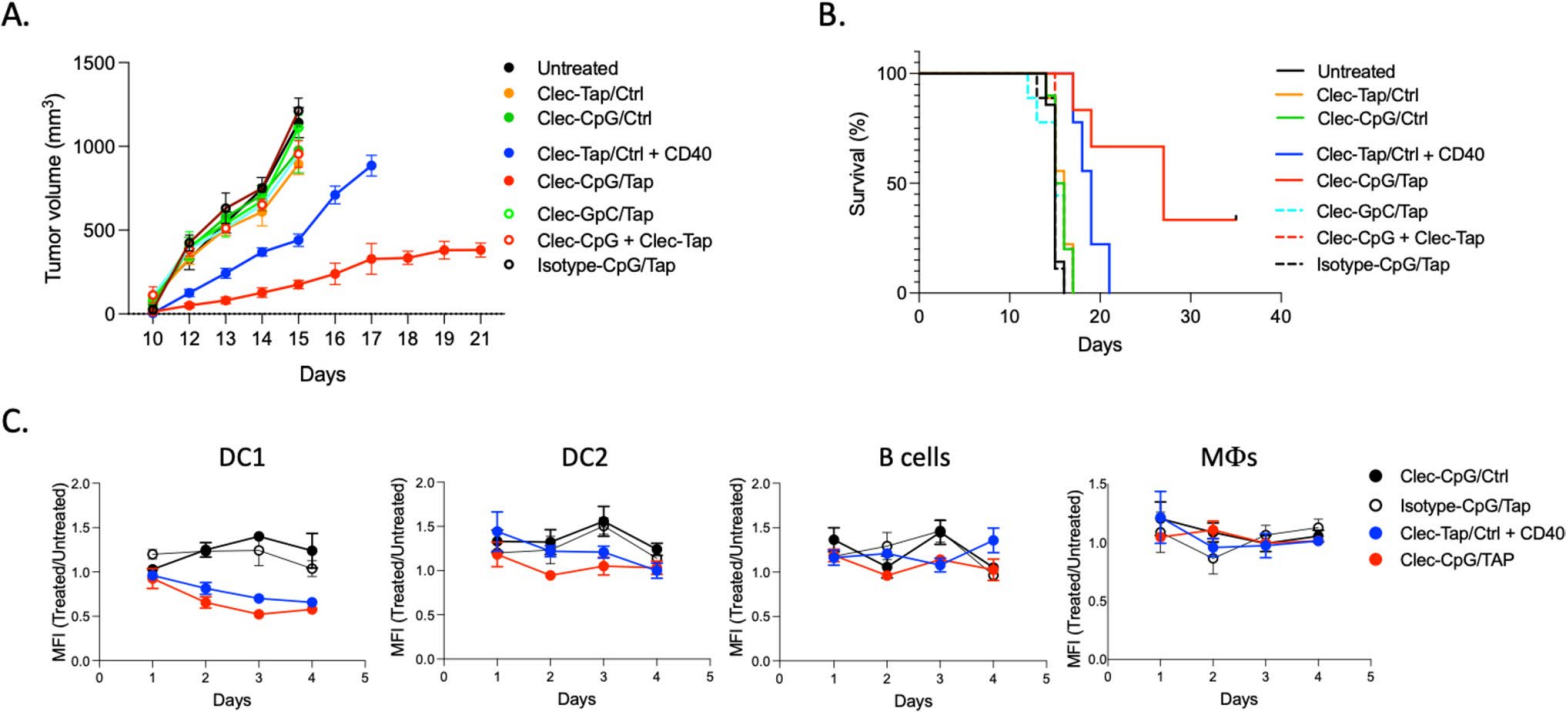
Treatment of TAP-deficient RMA-S tumor bearing mice with Clec9a antibody targeted TAP siRNA and CpG ODN. As in legends to Fig. 3. Clec9a-TAP siRNA and CpG ODNs were administered either separately (Clec9a-Tap + Cle9a-CpG) or on the same antibody (Clec9a-CpG/Tap). To maintain identical doses, when conjugated separately the TAP siRNA and CpG ODN were mixed with an equimolar amount of control siRNA or nonstimulatory GpC containing ODN prior to conjugation to Cle9a antibody. Isotype-CpG/Tap, TAP siRNA and CpG ODNs conjugated to a non-clec9a binding antibody. **a** Tumor growth in mice bearing subcutaneously implanted RMA-S tumors as described in Fig. 3. CpG-TAP versus CpG-Ctrl *p* < 0.01, Cle9-TAP versus Clec-Ctrl *p* < 0.005, Clec-TAP versus CpG-TAP *p* < 0.01. **b** Survival. CpG-TAP versus CpG-Ctrl p < 0.05, Clec-TAP versus CpG-TAP *p* < 0.01. **c** Downregulation of TAP in cDC1 isolated from the spleen of the treated mice, as described in Fig. 2

Figure 6a and b shows that treatment of mice bearing subcutaneously implanted RMA-S tumors with Clec9a antibody co-targeted TAP siRNA and CpG ODN (Clec-CpG/Tap) was more effective than treatment with Clec9a targeted TAP siRNA and CD40 antibody (Clec-Tap + CD40) and was dependent on co-delivery of the TAP siRNA and CpG ODN to the same cell because treatment with a mixture of Cle9a targeted TAP siRNA and Cle9a targeted CpG ODN (Clec-Tap siRNA + Clec-CpG) did not inhibit tumor growth. The ability of CpG ODNs to target DC (Figs. 3 and 4) did not contribute to tumor inhibition because an isotype antibody conjugated with TAP siRNA and CpG ODN (Isotype-CpG/Tap) was ineffective. A likely reason for the reduced antitumor activity of Cle9a-TAP siRNA + CD40 antibody seen in this experiment (Fig. 6b) compared to the experiment shown in Fig. 3b was that we used half the dose of TAP siRNA, 4 versus 8 siRNAs per antibody, respectively, in order to accommodate 4 control siRNAs on the Clec9a antibody.

To further examine the mechanism of action of the Clec9a Ab conjugated CpG ODN, we measured the downregulation of TAP isolated from mice as described in Fig. 2. Figure 6c shows that Clec9a targeted TAP siRNA (Clec-Tap/Ctrl + CD40 and Clec-CpG/Tap) led to robust and sustained downregulation of TAP in cDC1 but not B cells or macrophages. A small and transient downregulation of TAP was also seen in cDC2 that was not seen in the experiment shown in Fig. 2, perhaps reflecting low levels of Clec9a expression in this preparation. Notably, no TAP downregulation was seen in cDC1 when mice were treated with isotype antibody conjugated to both CpG ODN and TAP siRNA (Isotype-CpG/Tap). This is consistent with the immunotherapy experiment (Fig. 6a and b) showing that the antibody immobilized CpG ODN was not responsible for targeting the Clec9a bound TAP siRNA to DC. Taken together, Fig. 6 shows that co-targeting TAP siRNA and CpG ODNs to cDC1 leads to a superior antitumor response, presumably by limiting DC activation to cells also presenting the induced antigens.

## Discussion

Here, we describe a method to target a TAP specific siRNA to resident Clec9a expressing cross-presenting cDC1 cells in mice which led to inhibition of tumor growth, dispensing with the need to isolate and expand cDC1 in vitro. Targeting was achieved by conjugation of the TAP siRNA to a Clec9a antibody which leads to the presentation of new antigens and priming of an antitumor immune response. Several studies have used Clec9a antibody to target tumor specific antigens to cDC1 in mice [6–8]. A main advantage of the TAP inhibition approach is that it would be applicable to all cancer patients because TAP downregulation leads to the presentation of a common set of new antigens in all cells in which TAP expression is reduced [21], thereby obviating the need to develop tumor- or patient-specific antigen targeting formulations.

One limitation of Clec9a targeting is that unlike CpG ODN targeting it does not provide maturation stimuli to the targeted dendritic cells, which in this study was provided by systemic treatment with an agonistic CD40 Ab [18, 19]. To obviate the need of using a second drug formulation, we exploited the multivalent nature of this antibody platform to combine both the antigen inducing and DC maturation functions into one drug formulation that was accomplished by hybridizing a mixture of TAP siRNA and CpG ODN to the Clec9a antibody (Fig. 5). Thus, activation/maturation of DC by the CpG ODN will be largely limited to those cDC1 cells that also present the TAP siRNA induced antigens, consequently reducing the risk of toxicities elicited by systemically administered immune modulatory agents like agonistic CD40 antibodies [20] or CpG ODNs [22]. Co-delivery of TAP siRNA and CpG ODNs to cDC1 inhibited tumor growth and exhibited superior antitumor activity than delivery of TAP siRNA alone in combination with CD40 Ab (Fig. 6). The CpG ODN that was used in this study serves as a prototype of a DC activating agent that may not be directly applicable to human patients because of significant differences in TLR9 expression patterns between mice and humans [23].

Manipulating functions in resident cDC1 by targeted delivery of TAP siRNA and maturation stimuli to resident DC that lead to inhibition of tumor growth was demonstrated in this study using preclinical murine tumor models. To evaluate this approach in clinical settings, it will be necessary to develop the Clec9a antibody-TAP siRNA conjugates that recognize its human targets, and the maturation stimulus best suited for stimulating human DC and recapitulate the findings in vitro using human PBMC derived DC and CD8 + T cells.

A main current limitation of using antibodies as targeting ligands is the challenging nature of chemical conjugations that are often inefficient, require a purification process, and depend on instrumentation and skill sets that are not readily available. The Clec9a antibody targeting strategy described in this study is illustrative of a versatile and broadly applicable antibody-based targeting platform that overcomes the challenging nature of chemical conjugations. Conjugation of cell modulatory agents to the oligo-modified antibody carried out by a simple hybridization reaction (Fig. 1) is straightforward, efficient, and dispenses with the need for purification. The antibody platform is modular; the oligonucleotide modified antibody once prepared can be conjugated to diverse biological agents. Another useful feature of this antibody platform is its multivalent nature that enables the co-delivery of two or more cell modulatory agents conjugated to the antibody by hybridization of a mixture of two or more agents, and hence manipulate two or more functions in the targeted cells, illustrated in this study using a TAP siRNA and a CpG ODN that were co-delivered to cDC1 cells in vivo by conjugation to the Clec9a antibody (Figs. 5 and 6). While this study focused on a therapeutic application, this easy-to-use antibody platform can also serve as a versatile tool to probe the biology of specific cell subsets in vivo, for example using targeted delivery of RNAi to explore the role of cGAS/STING pathway, the role of IFN stimulated genes in cDC1, or the importance of cytotoxic versus cytokine mediated effector functions of CD8 + T cells.

### Supplementary Information

Below is the link to the electronic supplementary material.

Supplementary file1 (PDF 305 KB)

Supplementary file2 (DOCX 3372 KB)

## Data Availability

All data and materials in the main text and supplementary materials will be available to any researcher for purposes of reproducing or extending the finding described in this manuscript.

## Abbreviations

cDC1: Conventional type 1 dendritic cell
DC: Dendritic cell
MDSC: Myeloid-derived suppressive cells
Nucl: Nucleolin
ODN: Oligonucleotide
TLR9: Toll-like receptor 9
TAP: Transporter associated with antigen processing
TEIPP: T cell epitopes associated with impaired peptide processing

## References

[1] Merad M, Sathe P, Helft J, Miller J, Mortha A (2013). The dendritic cell lineage: ontogeny and function of dendritic cells and their subsets in the steady state and the inflamed setting. Annu Rev Immunol.

[2] Wculek SK, Cueto FJ, Mujal AM, Melero I, Krummel MF, Sancho D (2020). Dendritic cells in cancer immunology and immunotherapy. Nat Rev Immunol.

[3] Cabeza-Cabrerizo M, Cardoso A, Minutti CM, Pereira da Costa M, Reis e Sousa C (2021). Dendritic cells revisited. Annu Rev Immunol.

[4] Murphy TL, Murphy KM (2022). Dendritic cells in cancer immunology. Cell Mol Immunol.

[5] Garg AD, Coulie PG, Van den Eynde BJ, Agostinis P (2017). Integrating next-generation dendritic cell vaccines into the current cancer immunotherapy landscape. Trends Immunol.

[6] Lahoud MH, Radford KJ (2021) Enhancing the immunogenicity of cancer vaccines by harnessing CLEC9A. Hum Vaccin Immunother, pp 1–5. 10.1080/21645515.2021.1873056 PMC8920153 33625943

[7] Park HY, Tan PS, Kavishna R, Ker A, Lu J, Chan CEZ (2017). Enhancing vaccine antibody responses by targeting Clec9A on dendritic cells. NPJ Vaccines.

[8] Sancho D, Mourao-Sa D, Joffre OP, Schulz O, Rogers NC, Pennington DJ (2008). Tumor therapy in mice via antigen targeting to a novel. DC-Restricted C-type lectin J Clin Invest.

[9] Lahoud MH, Ahmet F, Kitsoulis S, Wan SS, Vremec D, Lee CN (2011). Targeting antigen to mouse dendritic cells via Clec9A induces potent CD4 T cell responses biased toward a follicular helper phenotype. J Immunol.

[10] Idoyaga J, Lubkin A, Fiorese C, Lahoud MH, Caminschi I, Huang Y (2011). Comparable T helper 1 (Th1) and CD8 T-cell immunity by targeting HIV gag p24 to CD8 dendritic cells within antibodies to Langerin, DEC205, and Clec9A. Proc Natl Acad Sci U S A.

[11] Fossum E, Tesfaye DY, Bobic S, Gudjonsson A, Braathen R, Lahoud MH (2020). Targeting antigens to different receptors on conventional Type 1 dendritic cells impacts the immune response. J Immunol.

[12] van Hall T, Wolpert EZ, van Veelen P, Laban S, van der Veer M, Roseboom M (2006). Selective cytotoxic T-lymphocyte targeting of tumor immune escape variants. Nat Med.

[13] Garrido G, Schrand B, Rabasa A, Levay A, D'Eramo F, Berezhnoy A (2019). Tumor-targeted silencing of the peptide transporter TAP induces potent antitumor immunity. Nat Commun.

[14] Garrido G, Schrand B, Levay A, Rabasa A, Ferrantella A, Da Silva DM (2020). Vaccination against nonmutated neoantigens induced in recurrent and future tumors. Cancer Immunol Res.

[15] Iwasaki A, Medzhitov R (2004). Toll-like receptor control of the adaptive immune responses. Nat Immunol.

[16] Kawasaki T, Kawai T (2019). Discrimination between self and non-self-nucleic acids by the innate immune system. Int Rev Cell Mol Biol.

[17] Karapetyan L, Luke JJ, Davar D (2020). Toll-like receptor 9 agonists in cancer. Onco Targets Ther.

[18] Bonifaz L, Bonnyay D, Mahnke K, Rivera M, Nussenzweig MC, Steinman RM (2002). Efficient targeting of protein antigen to the dendritic cell receptor DEC-205 in the steady state leads to antigen presentation on major histocompatibility complex class I products and peripheral CD8+ T cell tolerance. J Exp Med.

[19] Byrne KT, Vonderheide RH (2016). CD40 stimulation obviates innate sensors and drives T cell immunity in cancer. Cell Rep.

[20] Vonderheide RH (2020). CD40 agonist antibodies in cancer immunotherapy. Annu Rev Med.

[21] Marijt KA, Doorduijn EM, van Hall T (2019). TEIPP antigens for T-cell based immunotherapy of immune-edited HLA class I(low) cancers. Mol Immunol.

[22] Ribas A, Medina T, Kirkwood JM, Zakharia Y, Gonzalez R, Davar D (2021). Overcoming PD-1 blockade resistance with CpG-A toll-like receptor 9 agonist vidutolimod in patients with metastatic melanoma. Cancer Discov.

[23] Hartmann G (2017). Nucleic acid immunity. Adv Immunol.

