# Supplementary material for: Vaccination against neoantigens induced in cross-priming cDC1 in vivo
Source: Cancer Immunol Immunother. 2024 Jan 17;73(1):9. doi: 10.1007/s00262-023-03597-y (PMC10794404; doi:10.1007/s00262-023-03597-y)
Supplement: Supplementary file 2 — Supplementary file2 (DOCX 3372 KB) [file 262_2023_3597_MOESM2_ESM.docx]

**SUPPLEMENTARY MATERIAL**

**
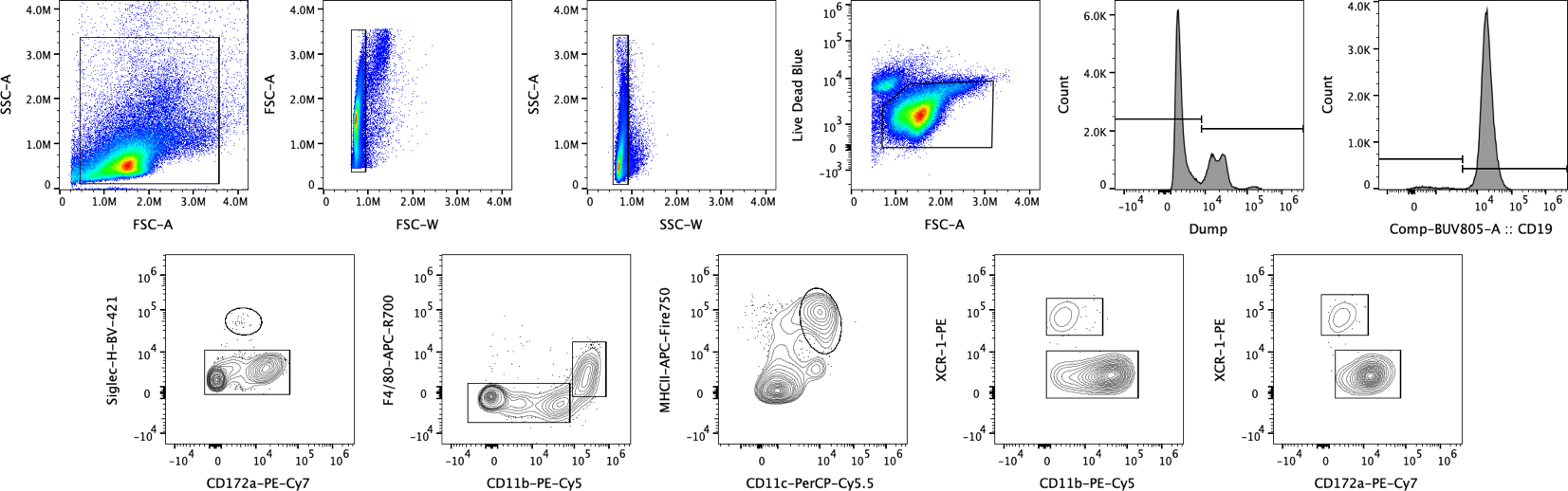
**

**Fig. S1.** Gating Strategy for Immune Cell Subsets shown in Figs. 2 and 6C)**.** Dump+ (CD3^+^ (T cells), CD49b^+^ (NK Cells), and Ly-6G+ (neutrophils)) were gated out of live, singlet splenocytes. Next, B cells (Dump-CD19+) were gated. Dump-CD19- were then gated based on Siglec-H expression: Siglec-H+ (pDC) and Siglec-H-. F4/80 was then used to gate macrophages (Macs; F4/80+). DCs were gated out of the F4/80- cells based on their high expression of MHCII and CD11c. XCR-1 and CD11b or XCR-1 and CD172a were then used to separate cDC1 (XCR-1+CD11b-CD172a-) from cDC2 (XCR-1-CD11b+CD172a+).

Summary:

B cells: (Dump-CD19+)

pDC: (Dump-CD19-SiglecH+)

Macrophages: (Dump-CD19-SiglecH-F4/80+)

cDC1: (Dump-CD19-SiglecH-F4/80-MHCII+CD11c+XCR+CD11b-CD172a-)

cDC2 : (Dump-CD19-SiglecH-F4/80-MHCII+CD11c+XCR-CD11b+CD172a+)
